# Efficacy and safety of platelet-rich plasma combined with hyaluronic acid versus platelet-rich plasma alone for knee osteoarthritis: a systematic review and meta-analysis

**DOI:** 10.1186/s13018-022-03398-6

**Published:** 2022-11-19

**Authors:** Qing Zhang, Tuodong Liu, Yuan Gu, Yongquan Gao, Jiangdong Ni

**Affiliations:** Department of Orthopaedics, The Second Xiangya Hospital, Central South University, No. 139 Renmin Street, Changsha, 410000 Hunan People’s Republic of China

**Keywords:** Platelet-rich plasma, Hyaluronic acid, Knee osteoarthritis, Meta-analysis

## Abstract

**Purpose:**

To systematically evaluate the curative efficacy and safety of platelet-rich plasma (PRP) combined with hyaluronic acid (HA) in the treatment of knee osteoarthritis (KOA), comparing with platelet-rich plasma alone.

**Methods:**

Cochrane Library, PubMed, China National Knowledge Infrastructure (CNKI) and Embase were searched for randomized controlled trials (RCTs) and cohort studies regarding the efficacy and safety of platelet-rich plasma (PRP) combined with hyaluronic acid (HA) in the treatment of knee osteoarthritis (KOA) comparing with platelet-rich plasma alone before January 15, 2022. The methodological quality of the ultimately included studies was assessed comprehensively, and meta-analysis was implemented using RevMan 5.3 software.

**Results:**

Thirteen articles (9 RCTs, 4 cohort studies), including 1118 patients, were covered. There was no significant difference between the PRP + HA therapy and PRP-alone therapy in VAS scores at 3 months, 6 months and 12 months, WOMAC total scores at 3 months and KOOS at 1 month and 6 months. Compared with PRP-alone therapy, PRP + HA therapy was associated with significantly better improvement in VAS scores at 1 month, WOMAC total scores at 6 months, KOOS at 3 months, IKDC scores at 6 months and Lequesne index scores at 3 and 6 months. However, the smallest treatment effect of VAS scores, WOMAC total scores, KOOS and IKDC scores did not exceed the minimum clinically important difference (MCID). However, PRP + HA therapy got a greater reduction in the rate of adverse events, compared with PRP-alone therapy.

**Conclusion:**

The results of this meta-analysis indicated that PRP + HA therapy was not found to be superior to PRP-alone therapy in pain relief and function improvement for patients with KOA. However, combined PRP with HA injections was generally safer than PRP injections alone, by assessing the incidence of adverse events.

**Supplementary Information:**

The online version contains supplementary material available at 10.1186/s13018-022-03398-6.

## Background

Knee arthritis (KOA) refers to a common joint disease characterized by knee cartilage degeneration and joint space stenosis [1]. Knee arthritis has been found as a vital cause of knee pain and progressive loss of joint function [2]. A number of methods have been suggested for treating KOA, which consist of pharmacological, non-pharmacological and surgical therapy [3]. However, there are no clear drugs or methods that have been proved to change the development process of KOA [4]. Intra-articular injection therapy is recognized as a feasible non-operative treatment of KOA and traditionally included hyaluronic acid (HA) or glucocorticoid as means of promoting joint lubrication, reducing joint inflammation, and reducing pain [5–7]. Recently, clinicians have treated KOA with intra-articular injections of hyaluronic acid (HA), platelet-rich plasma (PRP) and bone marrow concentrate (BMAC) [8].

Hyaluronic acid (HA) refers to a naturally occurring glycosaminoglycan found in synovial fluid that increases viscosity and lubricates joints. It has been proven to treat KOA through limiting inflammatory pathways and reducing pain of knee [9, 10]. The American College of Rheumatology (ACR) has recommended the injection of intra-articular HA in patients in 2012 [11].

Platelet-rich plasma (PRP) refers to a type of autologous blood extract, containing high concentration of platelets. It contains a wide variety of growth factors and other bioactive molecules, which are considered to control the aberrant inflammatory processes and thus to facilitate tissue healing [12]. Intra-articular injection of HA is not only expensive, but also has an unsatisfactory effect on inflammation. Even though there is a lack of definitive recommendations on PRP, the encouraging results reported by preliminary clinical evidences have allowed numerous clinicians to consider PRP as effective treatment for KOA [13].

Over the past few years, PRP + HA treatment has been progressively employed as a therapy of KOA. A number of articles have suggested that PRP + HA treatment might provide a synergistic effect, thus alleviating pain, ameliorating joint function and inhibiting the progress of KOA [14]. However, the number of articles, comparing the efficacy and safety of the combination therapy versus PRP-alone therapy, is limited. The aim of the meta-analysis was to systematically assess the curative efficacy and safety of PRP + HA therapy in the treatment of knee osteoarthritis (KOA), comparing with platelet-rich plasma alone.

## Methods

### Search strategy

This meta-analysis was following the Preferred Reporting Items for Systematic Review and Meta-Analysis statement (Additional file 1: PRISMA) [15]. Two independent researchers (QZ and YG) searched PubMed, EMBASE, Cochrane Library and CNKI up to January 15, 2022. Search terms were used including “platelet-rich plasma,” “hyaluronic acid,” “knee osteoarthritis.” The database retrieval strategies are illustrated in Additional file 2.

### Literature screening and data extraction

The relevant data from articles were abstracted by two researchers (TDL and YQG) independently using a prepared data extraction form. Information that was extracted from the selected studies consisted of publication year, authors, patient characteristics, study design, number of included patients, interventions, follow-up time, as well as outcomes. Any disagreement would be resolved through discussion with a third investigators.

### Risk of bias assessment

Two independent researchers (TDL and YQG) evaluate the risk of bias for RCTs by using the Cochrane Risk of Bias tool [16]. Each study was evaluated based on the following 7 areas: random sequence generation, allocation concealment, blinding of participants and personnel, blinding of outcome assessment, incomplete outcome data, selective reporting, as well as other biases. Two independent researchers (TDL and YQG) performed the quality assessment of cohort studies by using Newcastle–Ottawa Scale (NOS). The scale was divided into 3 items: selection, comparability, and exposure.

### Statistical analysis

RevMan 5.3 software was employed for analysis. Risk ratio (RR) with 95% confidence interval (95% CI) was used for the dichotomous variables and mean difference (MD) with 95% CI for the continuous variables. When *P* < 0.05, the difference would be significantly significant. Fixed effects model was adopted to analyze data if there was low heterogeneity (*p* > 0.10, *I*^2^ < 50%); otherwise, random effects model would be used. The pooled effect sizes were compared with their minimum clinically important differences (MCIDs), defined as the smallest difference perceived as importantly the average patient [17]. If treatment effects meet or exceed MCID, treatment should be changed unless side effects or costs are excessive [18].

### Inclusion and exclusion

#### Inclusion criteria

(1) Studies: RCTs or cohort studies were included (2) Population: patients diagnosed with KOA; (3) Intervention: PRP + HA therapy; Comparator: PRP treated alone; and (4) At least one of the outcome indicators, including Visual Analogue Scale (VAS) scores, Western Ontario and McMaster Universities Arthritis Index (WOMAC) total scores, Knee Injury and Osteoarthritis Outcome Scores (KOOSs), International Knee Documentation Committee (IKDC) scores, Lequesne index scores, as well as adverse events.

#### Exclusion criteria

(1) The studies that were not associated with treatment for KOA; (2) Laboratory or animal articles; (3) Duplicate publications, secondary publications or articles with similar data; and (4) Reviews, meeting abstracts, case reports, letters to the editor or commentaries.

## Results

### Literature searching

**Fig. 1:**
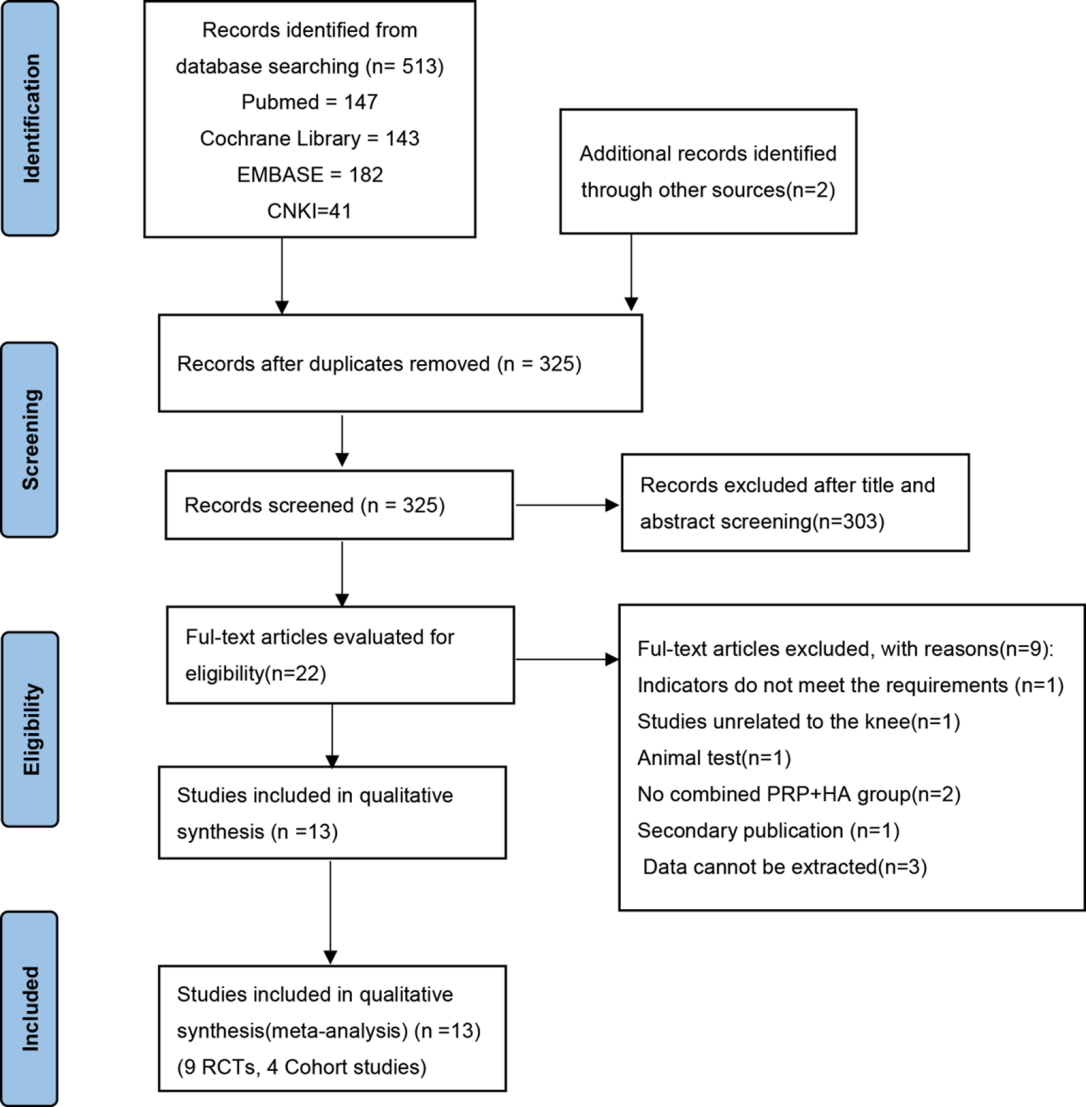
PRISMA search flow diagram (last search: January 2022)

A total of 515 related articles were identified. After eliminating duplicates, a total of 325 related articles initially identified. Then, 22 articles were retrieved for full text, after screening titles and abstracts. Nine RCTs [14, 19–26] and 4 cohort studies [27–30] were eventually included in this study. The above articles hold a total patient sample size of 1118. Figure 1 presents the article screening process and results.

Table 1 lists the basic characteristics of the covered articles. The ages of participants were primarily concentrated, ranging from 40 to 60 years. The Kellgren and Lawrence grading scales were I to IV. Follow-up time reported ranged from for 5 weeks to 24 months. There were some differences in the preparation of PRP among the included literatures, which may have affected the efficacy of KOA treatment. Additional file 3 presents the results.

**Table 1:** Important characteristics of patients enrolled in the included studies

Study, year	Study design	Sample size		Gender (M/F)		Age, years		Kellgren-Lawrence score (I:II:III:IV)		Clinical outcomes	Follow-up periods
		PRP + HA	PRP	PRP + HA	PRP	PRP + HA	PRP	PRP + HA	PRP		
Zhao [19]	RCT	62	62	35/27	37/25	55.73 ± 7.18	56.32 ± 8.13	–	–	①	5 weeks
Rao [20]	RCT	20	20	–	–	73.3 ± 7.2	73.3 ± 7.2	–	–	①	5 weeks
Ke [21]	RCT	50	50	25/25	24/26	57.80 ± 6.90	53.9 ± 7.1	5:11:20:14	6:12:20:12	②④⑤⑥	12 months
Ding [22]	RCT	20	27	2/18	8/19	56.75 ± 9.536	62.11 ± 12.50	9:6:5:0	10:11:6:0	①②,⑤⑥	6 months
Yu [14]	RCT	104	96	46/50	54/50	46.50 ± 7.50	46.20 ± 8.60	–	–	②	12 months
Jacob [23]	RCT	20	31	–	–	–	–	–	–	①/④	6 months
Sun [24]	RCT	39	39	21/18	17/22	60.6 ± 8.4	58.4 ± 8.1	0:39:0:0	0:39:0:0	①②⑥	6 months
Lana [25]	RCT	33	36	6/27	7/29	62 ± 6.1	60.9 ± 7	5:14:14:0	9:14:13:0	⑥	12 months
Xu [26]	RCT	48	40	–	–	57.9 ± 4.1	56.9 ± 4.2	0:25:23	0:19:21	⑥	24 months
Huang [27]	Co, P	31	33	8/23	8/25	63 ± 7.02	65.03 ± 7.10	0:10:10:11	0:9:15:9	③⑥	6 months
Guo [28]	Co, R	63	63	45/18	51/12	61.2 ± 9.6	60.7 ± 10.1	17:28:18:0	15:31:17:0	①②⑥	12 months
Abate [29]	Co, R	40	40	31/9	21/19	56.7 ± 11.2	60.90 ± 9.0	0:23:17:0	0:19:21:0	①③	6 months
Palco [30]	Co, R	28	23	12/16	12/11	62.71 ± 7.88	54.04 ± 10.4	0:8:20:0	0:10:13:0	①③	12 months

*RCT* randomized control trail, *Co* Cohort study, *P* prospective study, *R* retrospective study. *PRP* platelet-rich plasma, *HA* hyaluronic acid

① VAS scores, ② WOMAC total scores, ③ KOOS, ④ IKDC scores, ⑤ Lequesne index scores, ⑥ Adverse events

### Quality assessment of the included literature

**Fig. 2:**
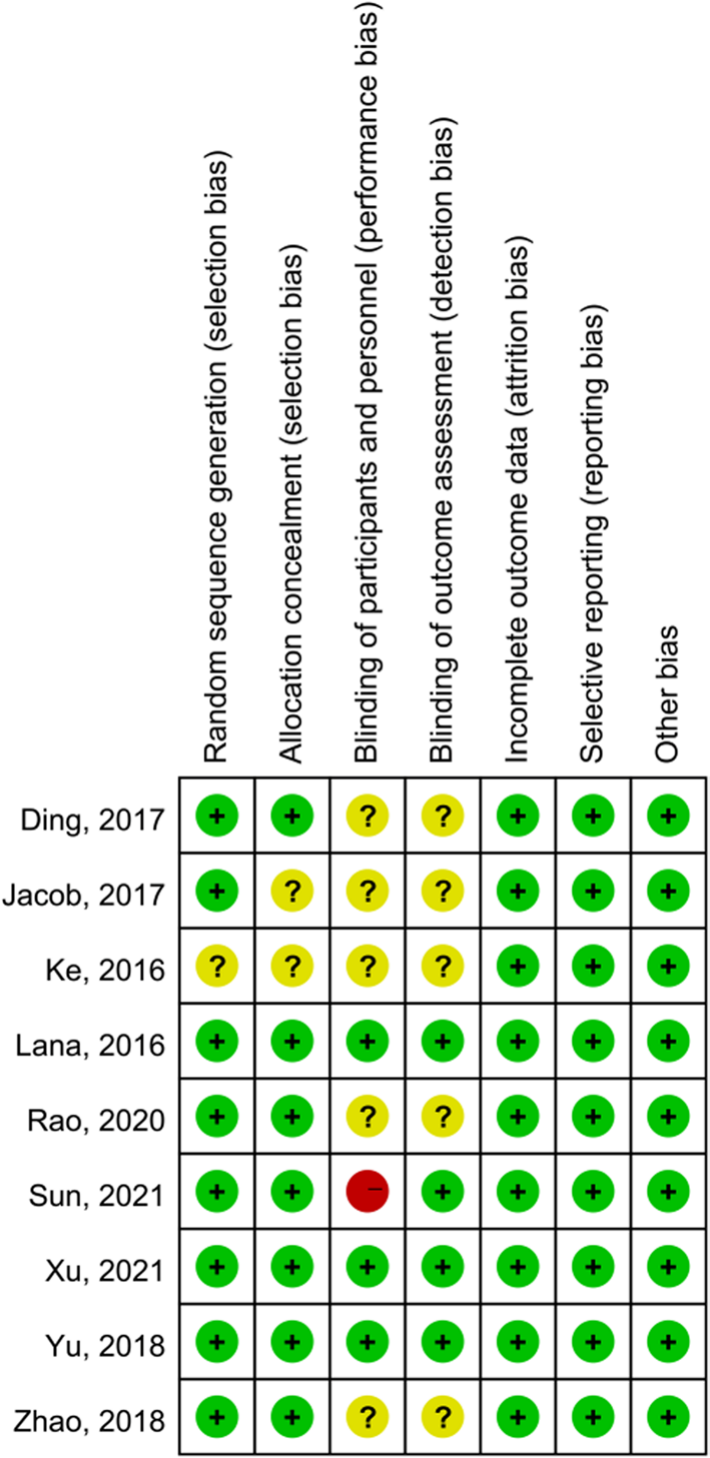
Risk of bias summary

**Fig. 3:**
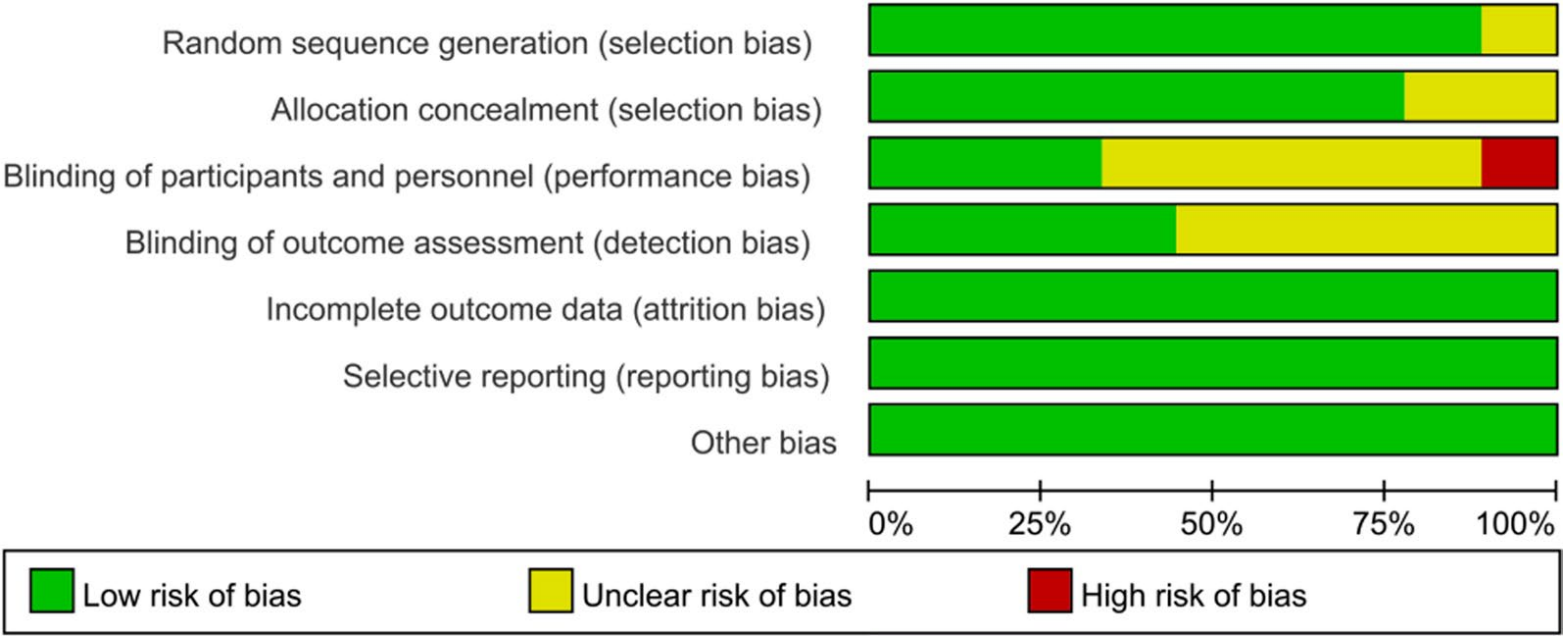
Risk of bias graph

Among the 9 RCTs, 8 articles explicitly reported the specific method of random assignment, and one study only mentioned randomness without specifying the specific method. Two out of 9 studies did not clarify the allocation concealment. For blinding of participants and personnel, the risk of bias was high in 1 out of 9 studies, was unclear in 5 out of 9 studies and was low in 3 out of 9 studies. For blinding of outcome assessment, the risk of bias was unclear in 5 out of 9 studies and was low in 4 out of 9 studies. For incomplete outcome data, selective reporting and other bias, all studies had low risk of bias. (Figs. 2 and 3). For cohort studies, the mean NOS for the 4 cohort studies was 8.25 (Table 2).

**Table 2:** Newcastle–Ottawa Scale for risk of bias assessment of cohort studies included in the meta-analysis

Study	Selection				Comparability	Outcome			Overall
	Representativeness of exposed cohort	Selection of non-exposed	Ascertainment of exposure	Outcome not present at start		Assessment of Outcome	Adequate Follow-Up Length	Adequacy of Follow-Up	
Huang [27]	★	★	★	★	★★	★	★		9
Guo [28]	★	★	★	★	★★	★	★	☆	8
Abate [29]	★	★	★	★	★★	★	★		9
Palco [30]	★	★	★	★	★	★	★	☆	7

### MCID

Based on previous work, the MCID for both the pain VAS scores and the WOMAC total scores was set at 20% [19–22]. Therefore, based on the baseline values of each outcome in the included studies, the MCID of the pain VAS scores was calculated to be 1.16, and the WOMAC total score was 7.86. In addition the mean difference between the two groups was compared with the MCID for each score reported in the literature: 11.5/100 for the IKDC scores [31] and 10/100 for the KOOS subscale [32].

### Outcomes of the meta-analysis

#### VAS scores

**Fig. 4:**
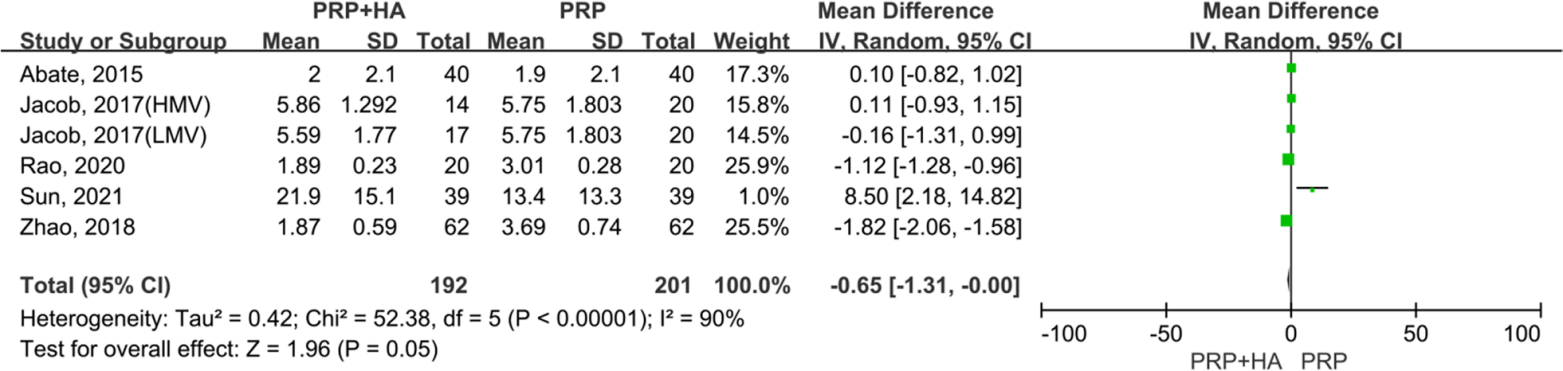
Forest plot and meta-analysis of VAS scores (1 month)

A total of 5 articles [19, 20, 23, 24, 29], including 393 patients, assessed VAS scores 1 month post-treatment. For the comparison of VAS scores at 1 month post-treatment between the experimental (PRP + HA therapy) group and the control (PRP-alone therapy) group, the smallest treatment effect (− 0.65) did not exceed the MCID (1.16). The analysis suggested that compared with the control group, the VAS scores of the experimental group at 1 month post-treatment were statistically significant, but not clinically significant (MD =  − 0.65, 95% CI − 1.31 to − 0.00, *p* = 0.05) (Fig. 4).

**Fig. 5:**
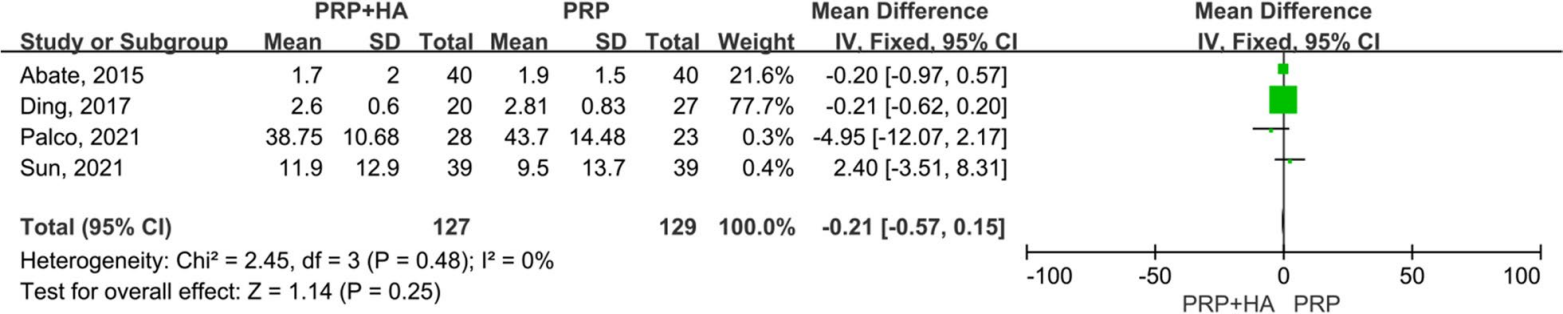
Forest plot and meta-analysis of VAS scores (3 months)

A total of 4 articles [22, 24, 29, 30], enrolling 256 patients, assessed VAS scores at 3 months post-treatment. For the comparison of VAS scores at 3 months post-treatment between the experimental (PRP + HA therapy) group and the control (PRP-alone therapy) group, the smallest treatment effect (− 0.21) did not exceed the MCID (1.16). The analysis suggested that the difference between the experimental (PRP + HA therapy) group and the control (PRP-alone therapy) group was neither statistically significant, but not clinically significant (MD =  − 0.21, 95% CI − 0.57 to 0.15, *p* = 0.25) (Fig. 5).

**Fig. 6:**
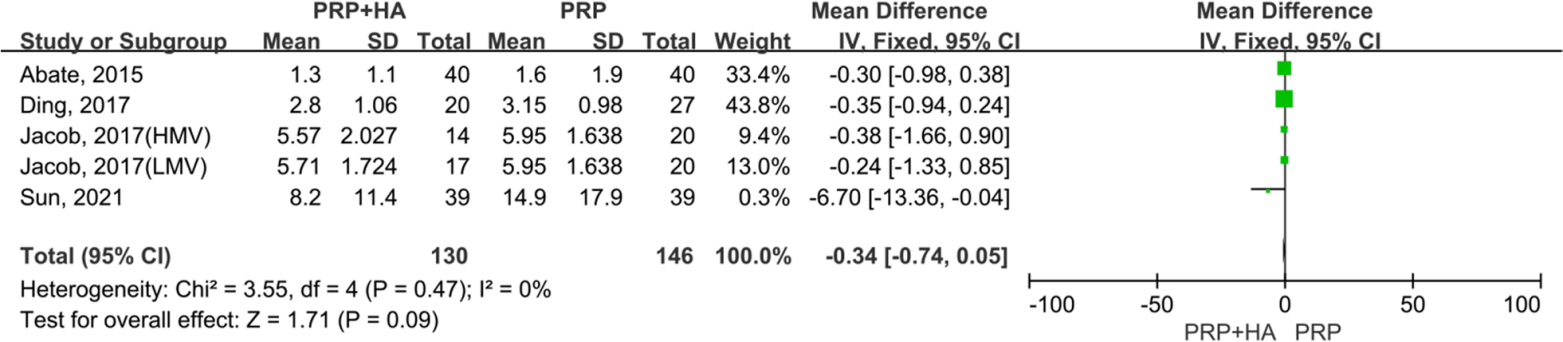
Forest plot and meta-analysis of VAS scores (6 months)

A total of 4 articles [22–24, 29], enrolling 276 patients, assessed VAS scores at 6 months post-treatment. For the comparison of VAS scores at 6 months post-treatment between the experimental (PRP + HA therapy) group and the control (PRP-alone therapy) group, the smallest treatment effect (− 0.34) did not exceed the MCID (1.16). The analysis suggested that the difference between the experimental (PRP + HA therapy) group and the control (PRP-alone therapy) group was neither statistically significant, but not clinically significant (MD =  − 0.34, 95% CI − 0.74 to 0.05, *p* = 0.09) (Fig. 6).

**Fig. 7:**
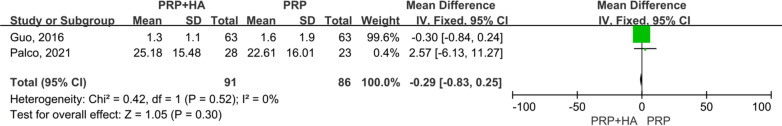
Forest plot and meta-analysis of VAS scores (12 months)

A total of 2 articles [28, 30], enrolling 177 patients, assessed VAS scores at 12 months post-treatment. For the comparison of VAS scores at 12 months post-treatment between the experimental (PRP + HA therapy) group and the control (PRP-alone therapy) group, the smallest treatment effect (− 0.29) did not exceed the MCID (1.16). The analysis suggested that the difference between the experimental (PRP + HA therapy) group and the control (PRP-alone therapy) group was neither statistically significant, but not clinically significant (MD =  − 0.29, 95% CI − 0.83 to 0.25, *p* = 0.30) (Fig. 7).

#### WOMAC total scores

**Fig. 8:**
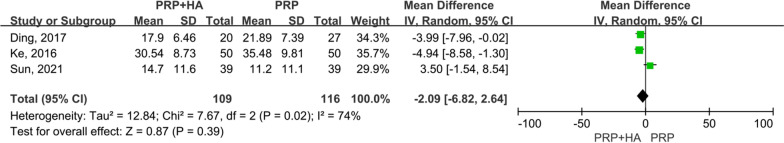
Forest plot and meta-analysis of WOMAC total scores (3 months)

A total of 3 articles [21, 22, 24], including 225 patients, assessed WOMAC total scores at 3 months post-treatment. For the comparison of WOMAC total scores at 3 months post-treatment between the experimental (PRP + HA therapy) group and the control (PRP-alone therapy) group, the smallest treatment effect (− 2.09) did not exceed the MCID (7.86). The analysis showed that compared with the control group, the WOMAC total scores of the experimental group at 3 months post-treatment was neither statistically significant, but not clinically significant (MD =  − 2.09, 95% CI − 6.82 to 2.64, *p* = 0.39) (Fig. 8).

**Fig. 9:**
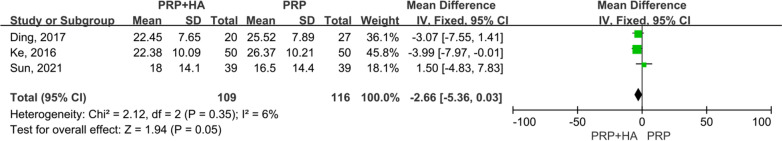
Forest plot and meta-analysis of WOMAC total scores (6 months)

A total of 3 articles [21, 22, 24], enrolling a total of 225 patient, assessed WOMAC total scores at 6 months post-treatment. For the comparison of WOMAC total scores at 6 months post-treatment between the experimental (PRP + HA therapy) group and the control (PRP-alone therapy) group, the smallest treatment effect (− 2.66) did not exceed the MCID (7.86). The analysis suggested that compared with the control group, the WOMAC total scores of the experimental group at 6 months post-treatment were statistically significant, but not clinically significant (MD =  − 2.66, 95% CI − 5.36 to 0.03, *p* = 0.05) (Fig. 9).

### KOOS

**Fig. 10:**
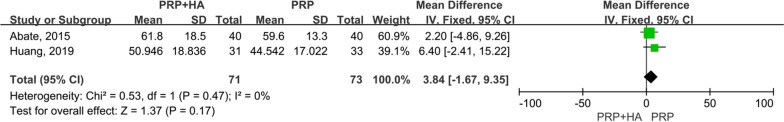
Forest plot and meta-analysis of KOOS (1 month)

Two articles [27, 29], including 144 patients, reported KOOS at 1 month after the treatment. For the comparison of KOOS at 1 month after the treatment between the experimental (PRP + HA therapy) group and the control (PRP-alone therapy) group, the smallest treatment effect (3.84) did not exceed the MCID (10). As revealed by the results, the difference between the experimental (PRP + HA therapy) group and the control (PRP-alone therapy) group was neither statistically significant, but not clinically significant (MD = 3.84, 95% CI − 1.67 to 9.35, *p* = 0.17) (Fig. 10).

**Fig. 11:**
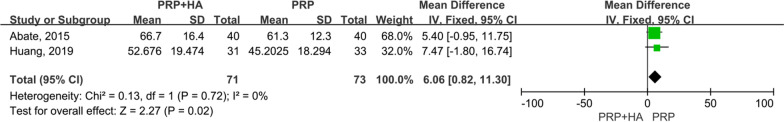
Forest plot and meta-analysis of KOOS (3 months)

Two articles [27, 29], enrolling 144 patients, reported KOOS at 3 months after the treatment. For the comparison of KOOS at 3 months after the treatment between the experimental (PRP + HA therapy) group and the control (PRP-alone therapy) group, the smallest treatment effect (6.06) did not exceed the MCID (10). The pooled results revealed that compared with the control group, the KOOS of the experimental group at 3 months post-treatment was statistically significant, but not clinically significant (MD = 6.06, 95% CI − 0.82 to 11.30, *p* = 0.02 < 0.05) (Fig. 11).

**Fig. 12:**
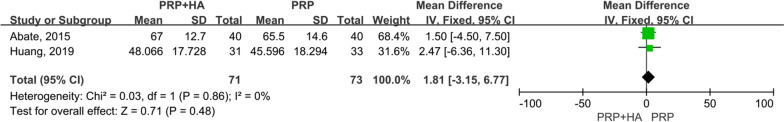
Forest plot and meta-analysis of KOOS (6 months)

KOOS was assessed by 2 articles [27, 29] in 144 patients at 6 months post-treatment. For the comparison of KOOS at 6 months after the treatment between the experimental (PRP + HA therapy) group and the control (PRP-alone therapy) group, the smallest treatment effect (1.81) did not exceed the MCID (10). The analysis suggested that the difference between the experimental (PRP + HA therapy) group and the control (PRP-alone therapy) group was neither statistically significant, but not clinically significant (MD = 1.81, 95% CI − 3.15 to 6.77, *p* = 0.48) (Fig. 12).

### IKDC scores

**Fig. 13:**
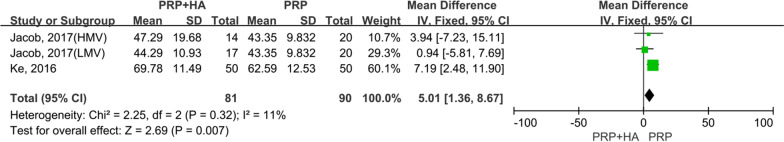
Forest plot and meta-analysis of IKDC scores (6 months)

IKDC scores were assessed 2 articles [21, 23] in 171 patients at 6 months post-treatment. For the comparison of IKDC scores at 6 months post-treatment between the experimental (PRP + HA therapy) group and the control (PRP-alone therapy) group, the smallest treatment effect (5.01) did not exceed the MCID (11.5). The results suggested that compared with the control group, the IKDC scores of the experimental group at 6 months post-treatment were statistically significant, but not clinically significant (MD = 5.01, 95% CI 1.36 to 8.67, *p* = 0.007 < 0.05) (Fig. 13).

### Lequesne index scores

**Fig. 14:**
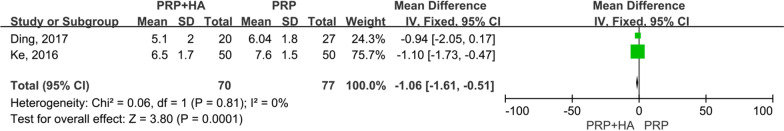
Forest plot and meta-analysis of Lequesne index scores (3 months)

A total of 2 articles [21, 22], including 147 patients, assessed Lequesne index scores at 3 months post-treatment. The analysis suggested that the scores of the experimental (PRP + HA therapy) group were lower than that of the control (PRP-alone therapy) group. (MD =  − 1.06, 95% CI − 1.61 to − 0.51, *p* = 0.0001 < 0.05) (Fig. 14).

**Fig. 15:**
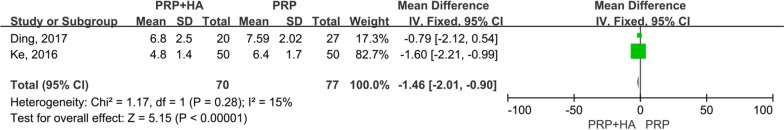
Forest plot and meta-analysis of Lequesne index scores (6 months)

Two articles [21, 22], including 147 patients, assessed Lequesne index scores at 6 months post-treatment. The analysis showed that the scores of the experimental (PRP + HA therapy) group were lower than that of the control (PRP-alone therapy) group (MD =  − 1.46, 95% CI − 2.01 to − 0.90, *p* = 0.00001 < 0.05) (Fig. 15).

### Adverse events

**Fig. 16:**
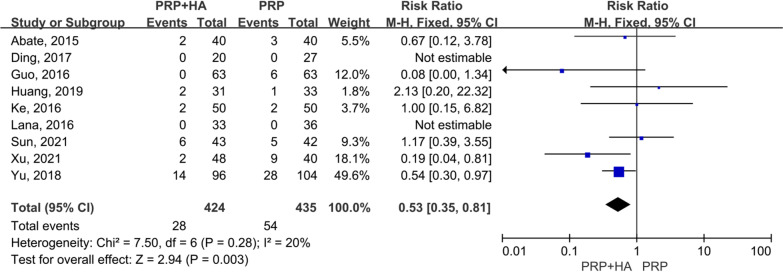
Forest plot and meta-analysis of Adverse events

Nine articles [14, 21, 22, 25–27, 29, 33, 34] with 859 patients reported the incidence of advance events. The pooled results demonstrated that in terms of adverse events, PRP + HA therapy was generally safer than PRP injections alone (RR = 0.53, 95% CI 0.35 to 0.81, *p* = 0.003 < 0.05) (Fig. 16).

## Discussion

KOA refers to one of the most common articular diseases, posing a serious hazard to the physical and mental health of patients. It has been recognized as the main cause of disability in the elderly [35]. Over the past few years, there have been a series of clinical trials comparing the effectiveness of different injections for treating KOA [36–38]. The majority of the above articles directly compared PRP versus HA, assessing their efficacy and safety using a considerable number of indicators, including VAS, WOMAC, or IKDC scores [39]. At present, there has been controversy relating to the clinical effect comparison between PRP and HA. There have been some articles concluding that PRP therapy could result in greater and longer-lasting improvements in alleviating pain, ameliorating the function of knee joint, in comparison with HA [37, 40]. However, Sun et al. [38]. concluded that the difference between PRP groups and HA groups in the clinical improvements of KOA was not significant. There have been numerous systematic reviews and meta-analyses, synthesizing preliminary studies and evaluating improvements in knee pain and function, generally confirming that PRP is beneficial for pain relief, and functional improvement, compared with HA and corticosteroids [41, 42].

During osteoarthritis, the synovium undergoes extensive changes, typically characterized by inflammatory pathology [43]. HA, a joint lubricant, has been extensively employed in clinical practice. HA has been confirmed to down-regulate pro-inflammatory factors (e.g., NFkB and PGE2), as well as proteases and proteinases known to break down the joint matrix [44]. HA is considered to be involved in proteoglycan and glycosaminoglycan synthesis, chondroprotection, as well as anti-inflammation [45]. Intra-articular injections of HA are capable of physically lubricating the articular surface, biologically nourishing articular cartilage, reducing wear of articular cartilage and facilitating endogenous HA synthesis, thus delaying further joint disease [45–47]. Intra-articular injection of PRP was considered to have the effect of alleviating knee pain, and ameliorating knee function with high safety [48]. PRP is capable of controlling the above inflammatory processes by releasing cytokines and growth factors [43]. PRP is capable of facilitating cell proliferation, collagen synthesis and vascularity, inducing a regenerative response and improving the metabolic function of damaged tissues [38, 49]. Over the past few years, the combination of PRP and HA has been considered to be more effective than PRP or HA alone in alleviating pain and ameliorating knee function. Razaq et al. suggested that PRP + HA therapy can exert a large synergistic effect on improving the outcome of moderate and severe KOA [50]. The combination therapy is capable of effectively relieving pain, improving function and reducing adverse reactions [26]. The synergistic effect is mainly through specific mediators (CD44, TGF-β1) to change the role of inflammatory cytokines in the degeneration of chondrocytes, so as to promote cartilage regeneration and inhibit inflammatory response [51]. The mechanism of this synergistic effect should be studied in depth.

There are some systematic reviews and meta-analyses, synthesizing primary studies and assessing improvements in pain and function. Zhao et al. found that compared with PRP alone, PRP + HA may have better clinical efficacy in the treatment of KOA [52]. Aw et al. found that compared with PRP alone, PRP + HA may be effective at providing pain relief and improvement in function up to 1 year following administration in the treatment of KOA [53]. However, there was a discrepancy between our conclusions and theirs. Over the past few decades, the concept of MCID has emerged in the outcome literature [54–57]. A clinically important difference is defined as a change or difference in an outcome measure deemed important and beneficial by the clinician or patient [17]. Our meta-analysis showed that the differences of VAS scores, WOMAC total scores, IKDC scores and KOOS between the two groups were less than their MCID values (MICD values: 1.16 for VAS scores, 7.86 for WOMAC total scores, 11.5 for IKDC scores and 10 for KOOS). Therefore, the statistical differences between the therapies found by VAS scores, WOMAC total scores, IKDC scores and KOOS were unlikely to have clinical significance. Although we found no literature on MCID for Lequesne index scores, based on the results of VAS scores, WOMAC total scores, KOOS, IKDC scores, we consider that PRP + HA therapy was not found to be superior to PRP-alone therapy in pain relief and function improvement for patients with KOA. We note that our conclusions differ from those of Zhao et al. and Aw et al. Probably because in this meta-analysis, we did not rely solely on statistical significance to measure the difference between the PRP + HA groups and the PRP-alone groups, we also used the MCID to help judge the clinical difference between the two groups.

However, some limitations were inevitable. Firstly, 4 articles were cohort studies, which may lead to the heterogeneity of the comprehensive index. Secondly, most of the articles had limited follow-up time, and the long-term curative efficacy and safety of PRP + HA therapy cannot be assessed. Thirdly, due to the small number of the selected studies, publication bias cannot be assessed by funnel plot. Fourthly, the concentrations of PRP used in the articles varied from study to study and may affect efficacy in the treatment of KOA.

## Conclusion

The results of this meta-analysis indicated that PRP + HA therapy were not found to be superior to PRP-alone therapy in pain relief and function improvement for patients with KOA. However, combined PRP with HA injections was generally safer than PRP injections alone, by assessing the incidence of adverse events.

Research registration Unique Identifying number (UIN)

1. Name of the registry: PROSPERO

2. Unique Identifying number or registration ID: CRD42022302539

3. Hyperlink to your specific registration (must be publicly accessible and will be checked): https://www.crd.york.ac.uk/prospero/display_record.php?RecordID=302539

## Supplementary Information

**Additional file 1.** PRISMA 2020 Checklist.

**Additional file 2.** Supplementary material 1.

**Additional file 3.** Supplementary material 2.

## Data Availability

All data generated or analyzed during this study are included in published articles.

## Abbreviations

PRP: Platelet-rich plasma
HA: Hyaluronic acid
KOA: Knee osteoarthritis
CNKI: China National Knowledge Infrastructure
RCT s: Randomized controlled trials
VAS: Visual analogue scale
WOMAC: Western Ontario and McMaster Universities Arthritis Index
KOOS: Knee Injury and Osteoarthritis Outcome Score
IKDC: International Knee Documentation Committee
MCID: Minimum clinically important difference
