# Supplementary material for: Efficacy and safety of platelet-rich plasma combined with hyaluronic acid versus platelet-rich plasma alone for knee osteoarthritis: a systematic review and meta-analysis
Source: J Orthop Surg Res. 2022 Nov 19;17:499. doi: 10.1186/s13018-022-03398-6 (PMC9675184; doi:10.1186/s13018-022-03398-6)
Supplement: Supplementary file 2 — Additional file 2. Supplementary material 1. [file 13018_2022_3398_MOESM2_ESM.docx]

Supplementary material 1

**Pubmed**

#1 ((Platelet-Rich Plasma[Title/Abstract]) OR (Plasma, Platelet-Rich[Title/Abstract])) OR (Platelet Rich Plasma[Title/Abstract])

#2 (((((((((((((Hyaluronic Acid[Title/Abstract]) OR (Acid, Hyaluronic[Title/Abstract])) OR (Amo Vitrax[Title/Abstract])) OR (Vitrax, Amo[Title/Abstract])) OR (Biolon[Title/Abstract])) OR (Etamucine[Title/Abstract])) OR (Hyaluronan[Title/Abstract])) OR (Hyvisc[Title/Abstract])) OR (Luronit[Title/Abstract])) OR (Sodium Hyaluronate[Title/Abstract])) OR (Hyaluronate, Sodium[Title/Abstract])) OR (Hyaluronate Sodium[Title/Abstract])) OR (Amvisc[Title/Abstract])) OR (Healon[Title/Abstract])

#3 ((((Osteoarthritis, Knee[Title/Abstract]) OR (Knee Osteoarthritides[Title/Abstract])) OR (Knee Osteoarthritis[Title/Abstract])) OR (Osteoarthritis of Knee[Title/Abstract])) OR (Osteoarthritis of the Knee[Title/Abstract])

#4 #1 AND #2 AND #3

#4  ((((Platelet-Rich Plasma[Title/Abstract]) OR (Plasma, Platelet-Rich[Title/Abstract])) OR (Platelet Rich Plasma[Title/Abstract])) AND (((((Osteoarthritis, Knee[Title/Abstract]) OR (Knee Osteoarthritides[Title/Abstract])) OR (Knee Osteoarthritis[Title/Abstract])) OR (Osteoarthritis of Knee[Title/Abstract])) OR (Osteoarthritis of the Knee[Title/Abstract]))) AND ((((((((((((((Hyaluronic Acid[Title/Abstract]) OR (Acid, Hyaluronic[Title/Abstract])) OR (Amo Vitrax[Title/Abstract])) OR (Vitrax, Amo[Title/Abstract])) OR (Biolon[Title/Abstract])) OR (Etamucine[Title/Abstract])) OR (Hyaluronan[Title/Abstract])) OR (Hyvisc[Title/Abstract])) OR (Luronit[Title/Abstract])) OR (Sodium Hyaluronate[Title/Abstract])) OR (Hyaluronate, Sodium[Title/Abstract])) OR (Hyaluronate Sodium[Title/Abstract])) OR (Amvisc[Title/Abstract])) OR (Healon[Title/Abstract]))Search results

**Embase**

#1 'Platelet-Rich Plasma':ab,ti or 'Plasma, Platelet-Rich':ab,ti or 'Platelet Rich Plasma':ab,ti

#2 'Hyaluronic Acid':ab,ti or 'Acid, Hyaluronic':ab,ti or 'Amo Vitrax':ab,ti or 'Vitrax, Amo':ab,ti or 'Biolon':ab,ti or 'Etamucine':ab,ti or 'Hyaluronan':ab,ti or 'Hyvisc':ab,ti or 'Luronit':ab,ti or 'Sodium Hyaluronate':ab,ti or 'Hyaluronate, Sodium':ab,ti or 'Hyaluronate Sodium':ab,ti or 'Amvisc':ab,ti or 'Healon':ab,ti

#3 'Osteoarthritis, Knee':ab,ti or 'Knee Osteoarthritides':ab,ti or 'Knee Osteoarthritis':ab,ti or 'Osteoarthritis of Knee':ab,ti or 'Osteoarthritis of the Knee':ab,ti

#4 #1 AND #2 AND #3

**Cochrane**

#1 (Platelet-Rich Plasma):ti,ab,kw OR (Plasma, Platelet-Rich):ti,ab,kw OR (Platelet Rich Plasma):ti,ab,kw

#2 (Hyaluronic Acid):ti,ab,kw OR (Acid, Hyaluronic):ti,ab,kw OR (Amo Vitrax):ti,ab,kw OR (Vitrax, Amo):ti,ab,kw OR (Biolon):ti,ab,kw OR (Etamucine):ti,ab,kw OR (Hyaluronan):ti,ab,kw OR (Hyvisc):ti,ab,kw OR (Luronit):ti,ab,kw OR (Sodium Hyaluronate):ti,ab,kw OR (Hyaluronate, Sodium):ti,ab,kw OR (Hyaluronate Sodium):ti,ab,kw OR (Amvisc):ti,ab,kw OR (Healon):ti,ab,kw

#3 (Osteoarthritis, Knee):ti,ab,kw OR (Knee Osteoarthritides):ti,ab,kw OR (Knee Osteoarthritis):ti,ab,kw OR (Osteoarthritis of Knee):ti,ab,kw OR (Osteoarthritis of the Knee):ti,ab,kw

#4 #1 AND #2 AND #3

**CNKI**

（透明质酸[摘要] OR 玻尿酸[摘要]）AND 富血小板血浆[摘要] AND（膝关节炎[主题] OR膝关节骨性关节炎[主题] OR膝骨关节炎[主题] OR膝关节骨关节病[主题] OR膝关节退行性关节炎[主题] OR膝关节骨关节炎[主题]）
