# Supplementary material for: Efficacy and safety of platelet-rich plasma combined with hyaluronic acid versus platelet-rich plasma alone for knee osteoarthritis: a systematic review and meta-analysis
Source: J Orthop Surg Res. 2022 Nov 19;17:499. doi: 10.1186/s13018-022-03398-6 (PMC9675184; doi:10.1186/s13018-022-03398-6)
Supplement: Supplementary file 3 — Additional file 3. Supplementary material 2. [file 13018_2022_3398_MOESM3_ESM.docx]

Supplementary material 2.

| Author, year | Preparation process | PRP+HA | PRP |
| --- | --- | --- | --- |
| Zhao, 2018 | PRP: collecting 5 mL of the patient's own whole blood and then centrifuging it to obtain PRP.  PRP and HA administered together. | 4 ml of PRP combined with 2.5 ml of HA, 1 time per week, 5 weeks. | 2 ml of PRP,  1 time per week, 5 weeks. |
| Rao, 2020 | PRP: 40 ml venous blood; centrifuged it; PCP (platelet-containing plasma) aspirated and transferred out, then centrifuged it again for 10 min; activation with 0.2 ml of calcium chloride; obtain 4 ml PRP.  PRP administered first, HA later. | 4 ml of PRP combined with 2 ml of HA,  1 time per week, 5 weeks. | 4 ml of PRP,  1 time per week, 5 weeks. |
| Ke, 2016 | PRP: 4 ml of sodium citrate anticoagulant added to 40 ml venous blood; centrifuged it at 1500 r/min for 10 min; PCP aspirated and transferred out, then centrifuged it again at 1500 r/min for 10 min; activation with 0.2 ml of calcium chloride; obtain 4 ml PRP.  PRP administered first, HA 10 min later. | 4 ml of PRP combined with 2 ml of HA, 1 time per week, 5 weeks. | 6 ml of PRP, 1 time per week, 5 weeks. |
| Huang, 2019 | PRP: 4 ml of sodium citrate anticoagulant added to 36 ml venous blood; centrifuged it; PCP aspirated and transferred out, then centrifuged it again; obtain 6 ml PRP.  PRP and HA administered together. | 6 ml of PRP combined with 2 ml of HA,  one time per two weeks, a total of three times. | 6 ml of PRP,  one time per two weeks, a total of three times. |
| Guo, 2016 | NS.  PRP and HA administered together. | NS.  1 time per week, 3weeks. | NS.  1 time per week,3weeks. |
| Ding, 2017 | PRP: sodium citrate anticoagulant added to 40 ml venous blood; centrifuged it at 1450 r/min for 10 min; PCP aspirated and transferred out, then centrifuged it again at 3370 r/min for 10 min; activation with 0.2 ml of calcium chloride; obtained 5 ml PRP.  PRP and HA administered together. | 4 ml of PRP combined with 2.5 ml of HA,  1 time per week, 3weeks | 4 ml of PRP,  1 time per week, 3weeks |
| Yu, 2018 | Plasma samples were prepared immediately using centrifugation at 2000 g at 4 °C for 10 min.  PRP and HA administered together | 8 ml of PRP combined with 0.2 ml of HA,  1 time per week, 8weeks | 8 ml of PRP  1 time per week, 8weeks. |
| Abate, 2015 | PRP+ HA: 4 ml of autologous blood added to 2 ml HA  20 mg/ml); centrifuged at 3500 rpm for 5 min; obtained 2 ml of PRP + 2 ml of HA.  PRP: 8 ml of whole blood, centrifuged at 3500 rpm for 5 min; obtained 4–5 ml of PRP.  PRP and HA administered together. | 2 ml of PRP combined with 2 ml of HA,  1 time per week, 3weeks. | 4–5 ml of PRP,  1 time per week, 3weeks |
| Jacob, 2017 | 20 ml sample of venous blood mixed with 5 ml of citrate phosphate dextrose solution. centrifuged at 3500 rpm for 7 min, PCP aspirated and transferred out, then centrifuged for at 3000 rpm for 5 min; obtained 2.5 ml of PRP.  PRP and HA administered together. | No detailed report. | 2ml of PRP. |
| Palco, 2021 | PRP + HA: Used RegenKit®-THT-3/RegenCell®(REGEN LAB SA, En Budron B2, 1052 Mont-sur-Lausanne, Switzerland) to obtain L-PRP and CellularMatrix A-CP-HA to obtain PRP + HA.  L-PRP: 8 ml of venous blood; centrifuged for 9 min at 3400 rpm/1500× g;  PRP + HA instead, was centrifuged for 5 min at 3400 rpm/1500× g.  PRP and HA administered together. | 3 ml of PRP combined with 2 ml of HA;  patients received 3 injections in 30 days (on the 1st, 15th, and 30th day). | 5 ml of PRP;  patients received 3 injections in 30 days (on the 1st, 15th, and 30th day). |
| Sun, 2021 | PRP:7 mL of venous blood was collected into a PLTenus PLUS Platelet Concentrate Separator (TCM Biotech International Corp., Taiwan); centrifuged at a speed of 500 ~ 1200 rpm for 8 min; obtained 3 mL of leukocyte-poor PRP.  HY AJOINT Plus is produced by microbial fermentation.  PRP and HA administered together. | 3 ml of PRP combined with 3 ml of HA,  No detailed report times, Intervals, and course of treatment. | 3 ml of PRP,  No detailed report No detailed report times, Intervals, and course of treatment. |
| Lana, 2016 | PRP: 8.5 ml of anticoagulant ACD (citric acid, sodium citrate, dextroset) added to 60 ml venous blood; centrifuged it at 300G for 5 min; PCP aspirated and transferred out, then centrifuged it again at 700G for 17 min; activation with 0.8 ml of thrombin; obtain 5 ml PRP.  PRP administered first, HA 10 min later. | 5 ml of PRP combined with 2 ml of HA,  one time per two weeks, a total of three times. | 5 ml of PRP,  one time per two weeks, a total of three times. |
| Xu, 2021 | PRP: 4ml of anticoagulant acid citate dextrose added to 36 ml venous blood; centrifuged it at 160 g for 10 min; PCP aspirated and transferred out, then centrifuged it again at 250 g for 15 min; obtain 5 ml PRP.  PRP and HA administered together. | 4 ml of PRP combined with 2 ml of HA,  a total of three injections, and the interval was half a month. | 4 ml of PRP,  a total of three injections, and the interval was half a month. |
